# Th17/Treg Cell Imbalance May Contribute to Spontaneous Preterm Labor

**DOI:** 10.1155/jimr/8405365

**Published:** 2025-05-22

**Authors:** Meiyi Xu, Cunling Zhang, Dan Wu, Liying Yao, Mengyuan Geng, Shanshan Li, Yuling Guo, Qiushui Wang, Zhuo Wei, Wen Li

**Affiliations:** ^1^Tianjin Institute of Gynecology Obstetrics, Tianjin Central Hospital of Gynecology Obstetrics, Tianjin, China; ^2^Tianjin Key Laboratory of Human Development and Reproductive Regulation, Tianjin Central Hospital of Gynecology Obstetrics, Tianjin, China; ^3^Tianjin Central Hospital of Gynecology Obstetrics, Nankai University, Tianjin, China; ^4^Institute of Analysis and Testing, Beijing Academy of Science and Technology (Beijing Center for Physical and Chemical Analysis), Beijing, China

**Keywords:** balance, spontaneous preterm labor, Th17, Treg

## Abstract

Spontaneous preterm labor (SPTL) is a major cause of neonatal mortality and severe complications. T cells play a crucial role in mediating inflammation and immune tolerance at the maternal–fetal interface. T helper 17 cells (Th17, pro-inflammatory) and regulatory T cells (Treg, anti-inflammatory) are two subsets of CD4^+^ T cells with opposite functions, and their balance is important for maintaining immune homeostasis. Since infection and inflammation represent prominent factors responsible for the pathogenesis of SPTL, Th17/Treg imbalance at the maternal–fetal interface may trigger proinflammatory responses, potentially leading to SPTL. In this review, evidence from both clinical cases of SPTL and animal models indicates the presence of Th17/Treg imbalance in both peripheral blood and the maternal–fetal interface. Additionally, interleukin-6 (IL-6), interleukin-1*β* (IL-1*β*), and interleukin-8 (IL-8) have been involved in the pathogenesis of inflammation-induced SPTL, suggesting that Th17/Treg imbalance may have relevance to and be involved in the pathogenic process of SPTL. Moreover, the presence of Th17/Treg imbalance in risk factors for SPTL, such as autoimmune diseases and bacterial infections, further supports this connection indirectly. Although predictive models and interventional strategies related to SPTL have been explored, there is currently insufficient evidence to establish a direct causal relationship between Th17/Treg imbalance and the onset of SPTL.

## 1. Introduction

Preterm birth accounts for ~5%–18% of all pregnancies. It is the leading cause of neonatal mortality and the second leading cause of mortality in children under the age of five. Preterm birth carries several risks and potential consequences for both the infant and the mother [1]. Of all singleton preterm births, ~65%–70% are due to spontaneous preterm labor (SPTL), whereas the remaining 30%–35% are a result of medically indicated preterm delivery [2]. Predicting and preventing SPTL remains challenging, often delaying timely management for high-risk patients [3]. Preterm labor (PTL) is a complex syndrome with multiple presumed etiologies, among which only acute pathological inflammation has been adequately characterized and causally linked to SPTL. A plethora of evidence from murine models and human studies now supports that SPTL is instigated by a breakdown in fetal–maternal tolerance coupled with excessive early inflammation [4].

While innate immune cells like macrophages and mast cells have been well studied in SPTL [5], T cells also have a crucial role in maintaining pregnancy through their abilities to induce immune homeostasis. T helper 1 (Th1) and T helper 2 (Th2) cell balance is believed to be the essential immune regulatory mechanism of T-cell immunology during pregnancy in the past [6]. However, regulatory T (Treg) and T helper 17 (Th17) cells being defined one after another, the mechanism of immune regulation between Treg and Th17 cells has been increasingly researched based on pregnancy.

Th17 and Treg are two subsets of CD4^+^ T cells with opposite actions. Th17 cells are a subset of pro-inflammatory T cells, while Treg cells are a subset of anti-inflammatory T cells. The balance between Th17 and Treg cells has an important role in maintaining immune homeostasis. Accumulated evidence suggests that Th17/Treg imbalance is involved in the development of various diseases, such as autoimmune and chronic infectious diseases [7]. In pregnancy outcomes, the association of the Th17/Treg balance with recurrent spontaneous abortions (RSAs) [8–10] and preeclampsia (PE) [11–13] has been established. Inflammation is a common underlying mechanism involved in SPTL [14], and Th17/Treg imbalance generally leads to an increased inflammatory response.

Therefore, in this review, we summarize the studies that have been conducted on the correlation between Th17/Treg imbalance and SPTL, as well as the associated regulatory factors, to provide a basis for a clearer understanding of the effect of the Th17/Treg imbalance in SPTL and insights into the future research directions concerning the correlation and mechanisms involving the imbalance and related cytokines in SPTL. Investigating the role of Th17/Treg imbalance in SPTL is instrumental in identifying early clinical predictive biomarkers and intervention targets for SPTL.

## 2. Th17/Treg Imbalance in SPTL

### 2.1. Peripheral Imbalance

Several studies have demonstrated that, compared to full-term deliveries, there is an imbalance of Th17/Treg in SPTL. This imbalance is primarily characterized by a significant reduction in the number of Treg cells [15–17]. Furthermore, the quantity of Treg cells is inversely correlated with both the gestational age at delivery and the severity of inflammation [16, 17]. This indicates that lower gestational ages and higher levels of inflammation are associated with a greater decline in Treg cell numbers. Additionally, peripheral blood samples from patients with SPTL exhibit lower expression of HLA-DR, a marker associated with the suppressive activity of Tregs. This suggests a significant impairment in the suppressive capacity of circulating Tregs in individuals with SPTL [18].

In a preterm mouse model using mifepristone (RU486), research has found an imbalance of Th17/Treg cells in the peripheral blood of preterm neonates. Specifically, compared to full-term neonates, preterm neonates exhibit an increase in Th17 cells and a decrease in Treg cells. This imbalance may, to some extent, reflect an inflammatory state in the mother [19]. Mouse studies also demonstrate that systemic depletion of Tregs triggers SPTL and results in adverse neonatal outcomes, whereas Treg supplementation prevents SPTL and improves neonatal prognosis [20].

### 2.2. Imbalance at the Maternal-Fetal Interface

Several studies have indicated that, compared to full-term controls, the levels of IL-17 or the number of Th17 cells are significantly increased in the amniotic fluid and at the maternal–fetal interface in cases of SPTL [21, 22]. This increase is particularly pronounced in SPTL cases complicated by chorioamnionitis (CAM), where a substantial recruitment of Th17 cells to the maternal–fetal interface is observed [23].

In animal models of SPTL precipitated by inflammatory processes, a plethora of investigations have consistently reported a perturbation in the equilibrium between Th17 and Treg at the maternal–fetal interface as well as in various systemic tissues. For example, within an equine model characterized by preterm delivery triggered by ascending placental inflammation, there was a marked increase in the prevalence of Th17 coupled with a concomitant reduction in Treg within the chorioallantois and endometrial compartments when juxtaposed with control specimens [24]. Analogous observations were made in a rhesus monkey model, wherein SPTL was induced by CAM, revealing an upsurge in Th17 and a diminution in Treg within the mediastinal, mesenteric lymph nodes, and the spleen relative to control groups [25]. Furthermore, in a murine model of SPTL elicited by lipopolysaccharide (LPS), a significant decrease in Treg was noted at the maternal–fetal interface in contrast to control conditions. Collectively, these observations underscore the pivotal role of Th17/Treg imbalance in the etiology of inflammation-associated SPTL.

## 3. Th17/Treg Imbalance and Risk Factors of SPTL

### 3.1. Autoimmune Disease

In autoimmune diseases such as systemic lupus erythematosus (SLE), rheumatoid arthritis (RA), and ankylosing spondylitis (AS), an imbalance between Th17 and Treg cells in peripheral blood is frequently observed, leading to systemic inflammation and autoimmunity [26]. The dysregulation of the Th17/Treg axis at local sites of inflammation is a hallmark of tissue-specific autoimmune pathogenesis, including conditions such as psoriasis, multiple sclerosis (MS), and crohn's disease. In these conditions, Th17 cells proliferate and dominate over Treg cells, exacerbating tissue damage [27, 28]. Therefore, autoimmune diseases are often associated with an imbalance of Th17/Treg cells in peripheral blood or affected local tissues.

Numerous studies have demonstrated that autoimmune diseases including SLE, RA, and psoriasis are increasingly recognized as significant risk factors for SPTL. Notably, the prevalence of SPTL in patients with SLE is ~13.3%, representing a 3.8-fold increased risk compared to healthy controls. Similarly, the prevalence in patients with RA is 7%, corresponding to a twofold increased risk. These elevated risks are likely attributed to the chronic inflammation and immune dysregulation characteristic of these autoimmune conditions [29, 30]. Therefore, it is reasonable to hypothesize that the imbalance of Th17/Treg cells in peripheral blood or local tissues observed in autoimmune diseases may be associated with, and potentially contribute to, the pathogenesis of SPTL.

### 3.2. Bacterial Infection

Studies have consistently shown that bacterial vaginosis (BV) is strongly associated with a nearly two-fold increased risk of SPTL, highlighting a significant correlation between this common vaginal infection and adverse pregnancy outcomes [31, 32]. Moreover, there is evidence that vaginal colonization with Group B streptococcus (GBS) is associated with an incidence of SPTL of ~22%, representing more than a twofold increased risk compared to noncolonized controls, underscoring the potential role of GBS colonization as a contributing factor to SPTL [33].

Furthermore, GBS in the reproductive tract can induce a significant Th17 response, which is associated with GBS colonization [34]. This indirectly demonstrates the correlation between Th17/Treg imbalance and SPTL.

Multiple cohort studies have consistently demonstrated that both clinical and subclinical CAM are strongly associated with a heightened incidence of SPTL [35, 36]. As previously mentioned, the recruitment of Th17 cells was observed in CAM [22]. Therefore, it is plausible to hypothesize that the elevated presence of Th17 cells in CAM may contribute to the increased risk of SPTL.

These associations suggest an Th17/Treg imbalance related to the infection might play a role in the inflammatory processes or immune responses that are involved in the pathogenesis of SPTL.

### 3.3. Maternal Comorbidities

Polycystic ovary syndrome (PCOS):

Women with PCOS exhibit approximately a twofold increased risk of SPTL compared to those without the condition [37]. Moreover, studies have demonstrated a significantly elevated Th17/Treg ratio in the peripheral blood of women with PCOS relative to healthy controls [38], suggesting that the heightened incidence of SPTL in this population may be associated with systemic Th17/Treg imbalance.

Gestational diabetes mellitus (GDM):

The incidence of SPTL among women with GDM is reported to be ~1.5 times higher than in normoglycemic pregnancies [39]. Notably, a significant reduction in the proportion of Treg cells in peripheral blood has been observed in GDM cases as early as the first trimester [40], along with evidence of impaired anti-inflammatory function of Treg cells [41]. Furthermore, studies have shown a marked increase in the peripheral Th17/Treg ratio in women with GDM at term [42], indicating that Th17/Treg imbalance may contribute to the elevated risk of SPTL observed in this group.

### 3.4. Additional Potential Risk Factors

Maternal psychological stress has been identified as a recognized risk factor for SPTL [43, 44]. However, there is currently no direct evidence linking elevated stress levels to Th17/Treg imbalance in either the peripheral circulation or the maternal–fetal interface. The only available evidence comes from animal studies, where chronic maternal stress was shown to induce Th17/Treg imbalance in the spleens of offspring [45].

Vaginal microbiome alterations have been associated with an increased risk of SPTL, with studies reporting a higher incidence of SPTL in women exhibiting a “low-Lactobacillus” vaginal microbiome profile [46]. Although some findings suggest that depletion of *Lactobacillus crispatus* may lead to elevated IL-17A levels in cervical and serum samples [47], studies directly investigating the correlation between reduced vaginal Lactobacillus levels and Th17 or Treg cell populations in either peripheral or local tissues remain lacking.

## 4. Factors Affecting the Th17/Treg Balance in SPTL

### 4.1. Interleukin-6

Interleukin-6 (IL-6) emerges as one of the most extensively studied cytokines among all cellular factors during pregnancy. Elevated levels of IL-6 are observed in amniotic fluid, maternal plasma, and cervical vaginal fluid during SPTL, underscoring its potential pivotal role in the pathogenesis of SPTL [48–50]. Furthermore, there is a marked increase in IL-6 levels upon microbial invasion of the amnion and chorion, as further evidenced in in vitro studies [51, 52]. Knockout of IL-6 in rodent models has been shown to prolong gestation, while treatment with recombinant IL-6 restores normal birth timing in these mice, underscoring the crucial role of IL-6 in the parturition process [53]. However, studies have revealed that high-dose intrauterine injection of IL-6 in rodent models does not induce SPTL [54]. Therefore, IL-6 may serve as a potential biomarker for SPTL and may be associated with the underlying mechanisms of SPTL. However, exposure to IL-6 alone is insufficient to induce SPTL.

IL-6 plays a pivotal role in determining the balance between Th17 cells and Treg cells. It inhibits the generation of Treg cells induced by TGF-*β* and promotes the differentiation of Th17 cells by suppressing TGF-*β*-induced Foxp3 expression and inducing ROR*γ*t [55, 56]. During CAM, there is a significant increase in Th17 cells, which is consistent with the elevated levels of IL-6 [57]. As previously mentioned, IL-6 promotes the development of Th17 cells while impairing the development of Treg cells, and this lymphocyte imbalance is associated with SPTL [58, 59].

Studies have shown that anti-IL-6R antibodies markedly decrease the occurrence of SPTL and neonatal mortality in LPS-induced murine inflammation models [60, 61]. These findings suggest that targeting IL-6 may mitigate SPTL and enhance neonatal outcomes by modulating the Th17/Treg balance.

### 4.2. Interleukin-1*β*

Interleukin-1*β* (IL-1*β*), the first known pro-inflammatory cytokine associated with SPTL, has been identified as a key inducer of SPTL inflammation through its binding to the IL-1R receptor [62]. It mediates the activation and amplification of the inflammatory cascade response [63]. The involvement of IL-1*β* as a cytokine in SPTL associated with intra-amniotic infection has long been reported [64]. Cases of SPTL with CAM exhibit higher expression levels of IL-1*β* in the amnion, chorion, and placenta compared to cases without CAM [65]. Intra-amniotic injection of IL-1*β* has been shown to induce CAM in rhesus monkeys, and infections/inflammations associated with CAM often lead to SPTL [25]. Moreover, IL-1*β* plays a crucial role in the pathogenesis of fetal systemic inflammation induced by CAM [66]. Therefore, IL-1*β* may serve as a biomarker for SPTL, and the inflammation induced by IL-1*β* is implicated in SPTL.

IL-1*β* is a potent inducer of Th17 differentiation, promoting the polarization of T cells towards the Th17 phenotype. IL-1*β* also plays a crucial role in promoting the secretion of IL-17 by T cells, as evidenced by the loss of IL-17 secretion capacity in T cells lacking IL-1R or in the presence of Caspase-1 (the enzyme required for IL-1*β* release) inhibition [67, 68]. Furthermore, IL-1*β* has been demonstrated to promote the conversion of Treg cells to the Th17 lineage, accompanied by downregulation of the transcription factor Foxp3 and suppression of Treg cell activity [69]. Collectively, these findings indicate that IL-1*β* possesses the capacity to increase the ratio of Th17 to Treg cells.

In rodent models, IL-1*β*-receptor inhibitors have been shown to attenuate SPTL induced by sterile IL-*β*-mediated inflammation or bacterial-like inflammation triggered by LPS [63]. These findings suggest that IL-1*β*-receptor inhibition may suppress SPTL by regulating the Th17/Treg imbalance.

### 4.3. Interleukin-8

Elevated levels of IL-8 have been observed in the amniotic fluid of cases with CAM compared to those without CAM [22]. Additionally, IL-8 levels in cervical–vaginal fluid have been positively correlated with SPTL [70], and they have been reported as predictive markers for SPTL and premature rupture of membranes [71, 72]. Moreover, bacterial-infected preterm neonates have significantly higher IL-8 levels in umbilical cord blood compared to noninfected preterm neonates [73]. These findings indicate an association between IL-8 and SPTL along with related inflammatory processes.

Research suggests that IL-8 can induce Th17/Treg imbalance in respiratory syncytial virus infection models [74]. Th17 cells at the maternal–fetal interface are positively correlated with IL-8 levels. While IL-17 alone does not enhance the secretion of IL-8 by amniotic mesenchymal cells, it can dose-dependently enhance IL-8 secretion induced by TNF-*α* [22]. While it remains unclear whether IL-8 mediates Th17/Treg imbalance in SPTL, it is evident that Th17 cells promote the production of IL-8 at the maternal–fetal interface.

### 4.4. Interleukin-10

IL-10 appears to exert a protective effect against endotoxin-induced PTL and fetal wastage in pregnant rats by reducing pro-inflammatory cytokines in the uterus and placenta [75]. However, in clinical cases of SPTL, the levels of IL-10 in amniotic fluid do not show significant changes compared to the control group [76, 77]. Despite the inhibitory role of IL-10 in inflammation observed in preterm animal models, no correlation between IL-10 and SPTL has been identified in clinical cases.

IL-10 secreted by Treg cells exerts inhibitory effects, preventing inflammation and autoimmune diseases [78, 79]. The presence of IL-10 promotes the expansion of inducible T regulatory (iTreg) cells and enhances their immunosuppressive function [80]. IL-10 can inhibit Th17 cell differentiation, Th17-related cytokines, and the expression of ROR*γ*t. Deletion of IL-10 in T cells leads to increased expression of IL-17, IL-22, and ROR*γ*t [81]. Therefore, Treg cells secrete IL-10 to exert anti-inflammatory effects, while IL-10 can suppress Th17 cell differentiation and responses. This implies that IL-10 may lower the Th17/Treg ratio, but to date, the correlation between IL-10 and SPTL remains unclear and requires further investigation.

## 5. Discussion

SPTL represents a profound threat to both maternal and neonatal health, with inflammatory processes and infectious agents identified as pivotal risk factors. It is hypothesized that the dysregulation of the Th17/Treg balance may be one of the key contributors to the pathogenesis of SPTL. In a subset of the studies reviewed, an observed Th17/Treg imbalance within both the peripheral blood and the maternal–fetal interface has been reported in cases of SPTL and in preterm animal models (Table 1), suggesting a potential link between this immunological disturbance and the disease's manifestation.

In vitro studies using human peripheral blood have demonstrated that Th17/Treg imbalance precedes the onset of SPTL [16]. Similarly, animal models have shown that Th17/Treg imbalance at the maternal–fetal interface also occurs prior to the manifestation of SPTL [24, 25], suggesting that such imbalance may contribute to the pathogenesis of SPTL. Moreover, evidence indicates that Th17/Treg imbalance is not only exclusive to SPTL but also occurs during term labor [17]. This raises the possibility that premature activation of Th17/Treg imbalance, potentially triggered by specific risk factors, may lead to SPTL. Given that autoimmune diseases, infections, and other established SPTL risk factors are frequently associated with Th17/Treg imbalance, it is reasonable to speculate that these factors may initiate the pathological cascade of SPTL through disruption of Th17/Treg homeostasis. Furthermore, commonly used animal models of SPTL often rely on these risk factors—such as infection or inflammation at the maternal–fetal interface—to induce PTL [23–25]. These models consistently show Th17/Treg imbalance preceding SPTL onset, further supporting the hypothesis that risk factor-induced Th17/Treg dysregulation plays a causative role in the development of SPTL.

Current clinical and experimental studies on SPTL still face several methodological limitations. Some human cohort studies have assessed the Th17/Treg balance in peripheral blood prior to the onset of SPTL. However, due to high experimental costs and the relatively low incidence of SPTL in the general population, it is impractical to conduct widespread screening for live Th17 and Treg cells during pregnancy. As a result, most human samples are obtained from individuals already exhibiting signs of SPTL, imposing significant constraints on both the timing of sample collection and the characteristics of the study population. These limitations hinder the ability to accurately evaluate the dynamic changes in Th17/Treg balance throughout the progression of SPTL, thereby restricting deeper investigation into its underlying immunological mechanisms. Animal models offer a means to address some of these challenges, as they allow for controlled induction of SPTL and flexible timing of immune cell analysis. However, common modeling approaches—such as induction of inflammation or infection at the maternal–fetal interface—fail to fully replicate the multifactorial etiology of human SPTL, limiting the translational relevance of these findings. Similarly, clinical assessments of Th17/Treg balance at the maternal–fetal interface are largely constrained by sample availability and are typically only feasible after SPTL has occurred, further restricting insights into immune mechanisms at this critical site. Although animal models allow for sampling prior to disease onset and may help overcome some limitations of human studies, their simplified and often singular approach to disease induction does not adequately capture the complexity of SPTL in humans. Consequently, interpretations of the immunopathological processes derived from such models may remain incomplete or skewed.

We conducted an in-depth analysis of the potential link between potential risk factors for SPTL and Th17/Treg cell imbalance, with a particular focus on autoimmune diseases, bacterial infections, and maternal comorbidities—all of which are associated with relative risks exceeding 1.5, and in some cases, approaching 2. Specifically, we examined whether these factors could directly induce Th17/Treg dysregulation. By synthesizing findings from multiple studies, we propose an indirect yet compelling hypothesis: disruption of Th17/Treg balance may play a critical role in the pathogenesis and progression of SPTL. Additionally, we considered two other recognized risk factors—stress and alterations in the microbiome. However, current evidence remains insufficient to establish a direct link between these factors and peripheral or local Th17/Treg imbalance, highlighting the need for further clinical investigations or animal studies to substantiate this potential connection.

In the context of SPTL, a multitude of cytokines, which serve as both biomarkers and regulatory elements, have been identified to exert influence on the equilibrium of the Th17/Treg (Table 2). These cytokines, encompassing a spectrum of pro-inflammatory and anti-inflammatory mediators, are integral to the complex immunological landscape that governs parturition timing and the maintenance of pregnancy. Among the pro-inflammatory mediators, IL-1*β* has been clearly demonstrated to induce SPTL in animal model experiments, whereas IL-6 and IL-8 individually have not shown the same effect. Whether these three cytokines can collectively promote the development of SPTL through amplification of the inflammatory cascade and participate in its pathogenic mechanisms remains to be elucidated through further in vitro and animal studies. Nonetheless, the three cytokines all play critical roles in driving the Th17/Treg imbalance. Our current understanding suggests that the cytokine-mediated modulation of the Th17/Treg imbalance may have profound implications for the risk of SPTL or contribute to the pathophysiological mechanisms leading to SPTL.

Preclinical studies have shown that adoptive transfer of Treg cells [20], administration of anti-IL-6 antibodies [61], and IL-1*β* inhibitors [63] can effectively prevent SPTL in animal models. However, these therapies have not yet entered clinical trials, possibly due to the high cost of large-scale application and the need for comprehensive safety evaluations. Further mechanistic research is warranted to support the development of novel interventions targeting the maintenance of Th17/Treg balance at both peripheral and maternal–fetal interface levels, with careful consideration of efficacy, safety, and cost-effectiveness. In addition, clinical cohort studies have demonstrated that vaginal administration of progesterone significantly increases the proportion of peripheral Treg cells [82], and in vitro experiments have shown that progesterone suppresses Th17 cell-mediated immune responses [83]. These findings suggest that progesterone may contribute to maintaining Th17/Treg balance. Progesterone has advanced to clinical trials for the prevention of SPTL, with multiple studies reporting a reduced incidence of SPTL following its use [84–86]. Notably, in 2011, the U.S. FDA approved progesterone for the prevention of recurrent preterm birth. However, due to substantial variability in its efficacy across different populations and obstetric histories [87], progesterone has not yet been widely adopted in routine clinical practice. Future large-scale, multicenter clinical trials with rigorous stratification by treatment route, population characteristics, and obstetric history are needed to determine whether progesterone can be broadly recommended for SPTL prevention.

**Figure 1:**
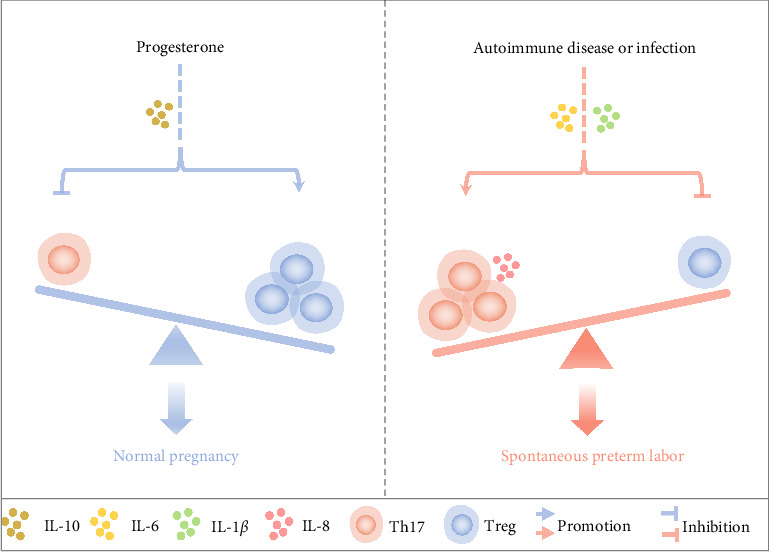
The proposed mechanism for the induction of SPTL by Th17/Treg imbalance is as follows: progesterone facilitates the differentiation of Treg cells and concurrently impedes the differentiation of Th17 cells within the peripheral blood or the maternal–fetal interface through IL-10 to maintain a healthy pregnancy. Conversely, autoimmune disease or infection triggers the production of factors IL-6 and IL-1*β*, leading to Th17 differentiation (promoting IL-8 secretion) and suppressing Treg differentiation, ultimately resulting in SPTL.

In this review, we thoroughly examine the complex relationship and proposed role of Th17/Treg imbalance in the etiology of SPTL (Figure 1). While the synthesis of the literature provides some direct evidence, it is important to acknowledge that much of our discussion is built on persuasive inferences drawn from existing research and well-established theoretical frameworks. Clarifying the potential impact of Th17/Treg imbalance in SPTL is a multifaceted endeavor that requires a comprehensive understanding of the underlying immunological dynamics. It is essential to recognize that the current evidence, while thought-provoking, is not conclusive, highlighting the need for more in-depth and detailed research into the immunological basis of SPTL.

To further elucidate the pathogenic mechanisms of Th17/Treg imbalance in SPTL, future research should focus on a deeper exploration of the underlying immune processes in SPTL. For instance, in vivo animal experiments could be employed to selectively knock out or replenish populations of Th17 and Treg cells, followed by statistical analysis of SPTL incidence and recovery rates. Alternatively, an SPTL animal model could be developed, and single-cell sequencing performed on PBMCs and maternal–fetal interface tissue-derived cells to identify differentially expressed genes associated with the Th17/Treg ratio. The functional roles of these genes in the pathogenesis of SPTL could then be validated through targeted in vitro and in vivo studies. Such detailed investigations will be instrumental in clarifying the specific role of Th17/Treg imbalance, thereby enhancing our comprehensive understanding of the disease's immunopathogenesis. In addition, to identify novel biomarkers, establish effective therapeutic targets, and ultimately advance early-stage treatment strategies for SPTL, a rigorously designed prospective clinical cohort study is essential. This study utilizes flow cytometry to analyze the Th17/Treg cell ratio in PBMCs of pregnant women across various gestational weeks, alongside ELISA to measure relevant cytokine levels in serum. These data are used to construct a predictive model for SPTL and assess the clinical utility of the Th17/Treg ratio and associated cytokines in predicting SPTL risk. Furthermore, the therapeutic potential of targeting these factors was explored through in vitro cell-based and in vivo animal experiments by modulating the Th17/Treg cell ratio or cytokine levels. These efforts provide a robust foundation for future clinical interventions, aiming to enable the early prevention and effective management of SPTL.

## Figures and Tables

**Table 1:** Literature review of Th17/Treg imbalance in SPTL.

References	Species	Sample size	Study design	Main experimental methods	Key findings	Organs	Limitations
[15]	Human	PTL (*n* = 6) Control (*n* = 18)	Clinical	FCM	Increased Th17/Treg cell ratio in the PTL group	Peripheral blood	Small experimental group size

[16]	Human	PTL (*n* = 36) Control (*n* = 15)	Clinical	FCM	Decreased Treg cell levels are correlated with a higher incidence of PTL	Peripheral blood	Sample selection bias: limited to women with PTL signs

[17]	Human	PTL (*n* = 15) Control (*n* = 20)	Clinical	FCM	Circulating Tregs reduced in PTL	Peripheral blood	Not specific to PTL, observed in both term and preterm deliveries

[18]	Human	PTL (*n* = 24) Control (*n* = 135)	Clinical	FCM	Reduction in the suppressive capacity of Tregs in PTL	Peripheral blood	Only functional decline observed; no change in proportion detected

[19]	Mouse	PTL (*n* = 15) Control (*n* = 15)	Experimental	FCM	Increased Th17/Treg cell ratio in premature mice	Peripheral blood	RU486-induced model may not fully replicate SPTL etiology

[22]	Human	PTL (*n* = 107) Control (*n* = 47)	Clinical	ELISA	Elevated amniotic fluid IL-17 in chorioamnionitis-associated PTL, derived from chorioamniotic Th17 cells	Maternal–fetal interface	Primarily observed in PTL with CAM; not generalizable to all PTL cases

[23]	Mouse	PTL (*n* = 7) Control (*n* = 10)	Experimental	FCM	Imbalance between Tregs and Th17 cells in the spleen in a model of PTL	Spleen	LPS-induced model may not fully replicate SPTL etiology

[24]	Equine	Placentitis (*n* = 7) Control (*n* = 10)	Experimental	IHC ELISA	Altered Th17/Treg balance at the maternal–fetal interface in the ascending placentitis model	Maternal–fetal interface	Placentitis model does not fully capture SPTL pathogenesis

[25]	Rhesus monkey	Chorioamnionitis (*n* = 10) Control (*n* = 7)	Experimental	FCM	Th17/Treg imbalance in lymphoid organs of the chorioamnionitis model	lymphoid organs	Chorioamnionitis model does not fully capture SPTL pathogenesis

Abbreviations: ELISA, enzyme-linked immunosorbent assay; FCM, flow cytometry; IHC, immunohistochemistry; PTL, preterm labor; SPTL, spontaneous preterm labor.

**Table 2:** Summary of factor effects.

Cytokines	Factors associated with SPTL		Factors affecting the Th17/Treg balance	
	Human (in vitro)	Animal models (in vivo)	Th17	Treg
IL-6	Amniotic fluid, maternal plasma, cervical vaginal fluid [48–50]	Mice: IL-6 shorten the gestation [53]	Promotes polarization (mice, in vitro) [55, 56]	Inhibits differentiation (mice, in vitro) [55, 56]
IL-1*β*	Amnion, chorion, placenta [65]	Rhesus monkeys: IL-1*β* can induce SPTL [25]	Promotes polarization (human and mice, in vitro) [67, 68]	Inhibit differentiation and activity (human, in vitro) [69]
IL-8	Amniotic fluid, cervical–vaginal fluid [22, 71]	—	Promotes differentiation (human, in vitro) [75]	—
IL-10	—	Rats: IL-10 exerts a protective role in SPTL [76]	Inhibits differentiation (mice, in vitro) [82]	Promotes differentiation and activity (human, in vitro) [81]

Abbreviation: SPTL, spontaneous preterm labor.

## Data Availability

Data sharing is not applicable to this article as no datasets were generated or analyzed during the current study.
